# Convergent Genomic Signatures of Cashmere Traits: Evidence for Natural and Artificial Selection

**DOI:** 10.3390/ijms24021165

**Published:** 2023-01-06

**Authors:** Wei Wang, Zhuohui Li, Guoxiang Xie, Xinmei Li, Zhipei Wu, Manman Li, Anguo Liu, Yan Xiong, Yu Wang

**Affiliations:** 1 Key Laboratory of Animal Genetics, Breeding and Reproduction of Shaanxi Province, College of Animal Science and Technology, Northwest A&F University, Yangling 712100, China; 2 College of Animal & Veterinary Sciences, Southwest Minzu University, Chengdu 610041, China

**Keywords:** cashmere traits, comparative genomics, convergent evolution, selection signatures, coexpression genes

## Abstract

Convergent evolution provides powerful opportunities to investigate the genetic basis of complex traits. The Tibetan antelope (*Pantholops hodgsonii*) and Siberian ibex (*Capra sibirica*) belong to different subfamilies in *Bovidae*, but both have evolved similar superfine cashmere characteristics to meet the cold temperature in plateau environments. The cashmere traits of cashmere goats underwent strong artificial selection, and some traces of domestication also remained in the genome. Hence, we investigated the convergent genomic signatures of cashmere traits between natural and artificial selection. We compared the patterns of convergent molecular evolution between Tibetan antelope and Siberian ibex by testing positively selected genes, rapidly evolving genes and convergent amino acid substitutions. In addition, we analyzed the selected genomic features of cashmere goats under artificial selection using whole-genome resequencing data, and skin transcriptome data of cashmere goats were also used to focus on the genes involved in regulating cashmere traits. We found that molecular convergent events were very rare, but natural and artificial selection genes were convergent enriched in similar functional pathways (e.g., ECM-receptor interaction, focal adhesion, PI3K-Akt signaling pathway) in a variety of gene sets. Type IV collagen family genes (*COL4A2*, *COL4A4*, *COL4A5*, *COL6A5*, *COL6A6*) and integrin family genes (*ITGA2*, *ITGA4*, *ITGA9*, *ITGB8*) may be important candidate genes for cashmere formation and development. Our results provide a comprehensive approach and perspective for exploring cashmere traits and offer a valuable reference for subsequent in-depth research on the molecular mechanisms regulating cashmere development and fineness.

## 1. Introduction

When species independently adapt to a new environment, the strong selection pressure sometimes leads to the independent evolution of similar phenotypic traits in different lineages [1,2]. Convergent evolution highlights that adaptive phenotypic convergence often results from genetic convergence in distant lineages [2,3]. With the development of genome sequencing technology, it provides new opportunities to explore the genetic mechanism of adaptive convergence. For instance, aquatic adaptation [4], high-elevation adaptation [5], desert adaptation [6], subterranean adaptation [7] and Arctic adaptation [8]. However, such convergent molecular events are still rare in nature. The Tibetan antelope (*Pantholops hodgsonii*) is endemic to living in the extremely high-altitude environment of the Qinghai-Tibetan Plateau, a cold region with low oxygen [9]. The Siberian ibex (*Capra sibirica*) is distributed throughout Tian-Shan, Pamir, Himalaya, Karakorum, Altai, and the mountains of Southern Siberia and Mongolia [10,11], with an average elevation of more than 3000 m; it has a typical cold climate [12]. The Tibetan antelope belongs to the family *Pantholopinae*, whereas the Siberian ibex belongs to the family *Capridae* [13]. Interestingly, due to these two species living in similar high-altitude, frigid environments, they co-evolved superfine cashmere features to withstand the cold. The fine fibers produced by the Tibetan antelope are called Shahtoosh, which is interpreted as “king of wool” in Persia, with an average fiber diameter of 10–12 μm [14]. Siberian ibex fibers (known as “Yangir” or “wild cashmere”) are also a high-grade textile raw material with an average fiber diameter of 13.6 μm and are widely traded as a substitute for Shahtoosh [15,16]. Obviously, Shahtoosh and Yangir, which are formed in the natural environment, have a finer diameter and better quality than artificially bred goat cashmere fiber (with an average fiber diameter of about 15 μm) [17,18]. Therefore, the current study focus on the convergent evolution characteristics of Tibetan antelope and Siberian ibex in cashmere traits not only provides a useful case for convergent evolution events but also helps to break the bottleneck of cashmere goat breeding. The goat (*Capra hircus*) is believed to be one of the first livestock species that underwent domestication approximately 10,000 years ago [19]. A variety of natural or artificial factors (e.g., environmental changes, human migration, and production-oriented breeding) have shaped the phenotypic diversity of goats [20]. Cashmere is an important production trait of goats with a long history of artificial selection during domestication; it is possible that decreasing temperatures motivated an increase in artificial selection for cashmere production in the cooler northern parts of China [21]. In previous research, studies on cashmere traits of cashmere goats mainly focus on phenotypic detection [22], genome-wide association studies [23], selection signal analysis [24] and skin transcriptome analysis [25]. However, there are limited reports on integrating multi-omics data to study cashmere traits, especially using comparative genomics.

In this study, we explored the convergent genomic signatures of natural and artificial selection of cashmere traits. Convergent positively selected genes, rapidly evolving genes, and amino acid substitutions were identified in Tibetan antelope and Siberian ibex based on 30 ruminant species’ genomic data. In order to focus convergently genomic features on cashmere traits, we also collected 156 whole-genome resequencing data of different species of goats (including 39 cashmere goats) to explore the selected genes of cashmere goats during artificial breeding. In addition, we integrated skin transcriptome data from 58 goats to assess the expression patterns of genes involved in regulating cashmere traits. We found that the convergent genes and sites of natural and artificial selection were rare but enriched in the same signaling pathway level. Our results provide insights into the genetic basis of convergent cashmere traits, which may be helpful for improving cashmere quality and broadening the breeding limit of cashmere goats.

## 2. Results

### 2.1. Genetic Evolution under Natural Selection Pressure

**Figure 1:**
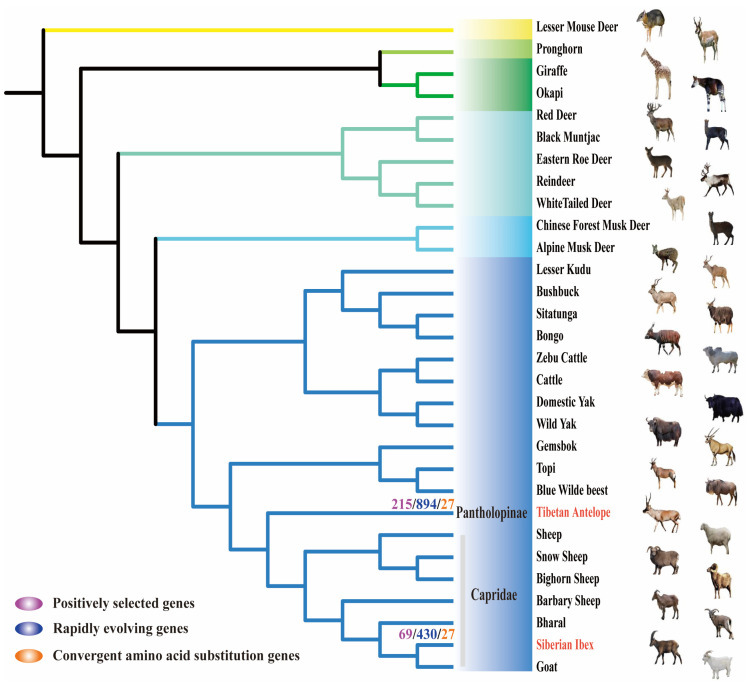
Species phylogenetic tree topology used for selection tests and inference of convergent evolution. The maximum likelihood phylogenetic tree of 30 ruminant species. The Lesser mouse deer is an outgroup. Different branch colors represent different families (including *Tragulidae*, *Antilocapridae*, *Giraffidae*, *Cervidae*, *Moschidae* and *Bovidae*).

**Figure 2:**
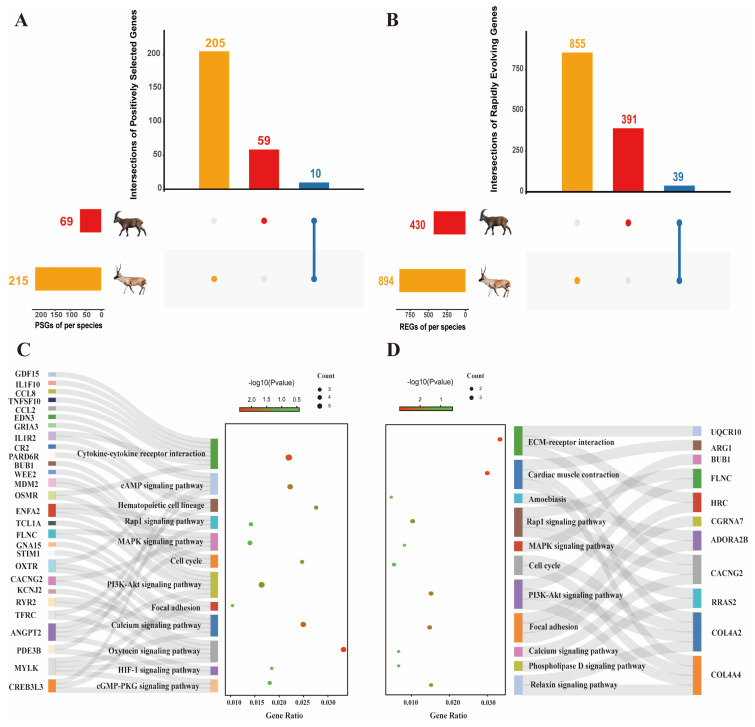
Analysis of positively selected genes and rapidly evolving genes between Tibetan antelope and Siberian ibex. (**A**) The number and overlap of PSGs in Tibetan antelope and Siberian ibex. (**B**) The number and overlap of REGs in Tibetan antelope and Siberian ibex. Orange column represents the uniqueness of Tibetan antelope, red column represents the uniqueness of Siberian ibex, blue column represents the commonality between the two species. (**C**) The KEGG pathway enrichment analysis of PSGs in Tibetan antelope. (**D**) The KEGG pathway enrichment analysis of PSGs in Siberian ibex.

In natural selection, similar environmental selection pressure often leads to adaptive phenotypic convergence, and the mechanism of phenotypic convergence is genetic convergence. To assess the selective pressures acting on Tibetan antelope (*Pantholops hodgsonii*) and Siberian ibex (*Capra sibirica*) in similarly superfine cashmere traits at the gene level, a phylogenetic tree of 30 ruminant species was constructed based on the 14,013 orthologs genes (Figure 1). Across these orthologs genes, we estimated the lineage-specific dN/dS ratio (ω) of each branch (codeml, a free ratio model). Then, we identified 215 and 69 positively selected genes (PSGs) (χ^2^ tests, *p* < 0.05) in the Tibetan antelope and Siberian ibex branch, respectively, using the branch-site model (Figure 2A and Tables S5 and S6). Similarly, we obtained 894 and 430 rapidly evolving genes (REGs) (χ^2^ tests, *p* < 0.05), respectively, using the branch model (Figure 2B and Tables S7 and S8).

To evaluate whether the same selective pressures act on the same genes and pathways, we compared the PSGs and REGs of each Tibetan antelope and Siberian ibex lineage. We found that 10 PSGs and 39 REGs were shared in two lineages (Figure 2A,B). For each set of genes identified as being under selective pressures, we performed a KEGG pathway enrichment analysis. The PSGs enriched results showed that several pathways, for instance, focal adhesion, calcium signaling pathway, PI3K-Akt signaling pathway, cell cycle and MAPK signaling pathway, were shared in Tibetan antelope and Siberian ibex (Figure 2C,D and Tables S9 and S10). However, we found limited evidence that shared pathway terms in Tibetan antelope and Siberian ibex were focused on the same selected genes. Only a single gene, *FLNC* (filamin C), was a shared PSG and commonly enriched in the focal adhesion pathway. Mutations in this gene have been linked to cardiac diseases such as arrhythmias [26], which may be related to the high-altitude adaptation and excellent running ability of Tibetan antelope and Siberian ibex. In addition, we also enriched the REGs and found that focal adhesion and PI3K-Akt signaling pathway were shared in Tibetan antelope and Siberian ibex. However, none of the same genes enriched in these shared pathways (Figure S2A,B, and Tables S11 and S12). These results suggest that the selective pressure may act on different genes but with similar functional pathways in adaptive evolution.

### 2.2. Signals of Convergent Evolution between Tibetan Antelope and Siberian Ibex

**Figure 3:**
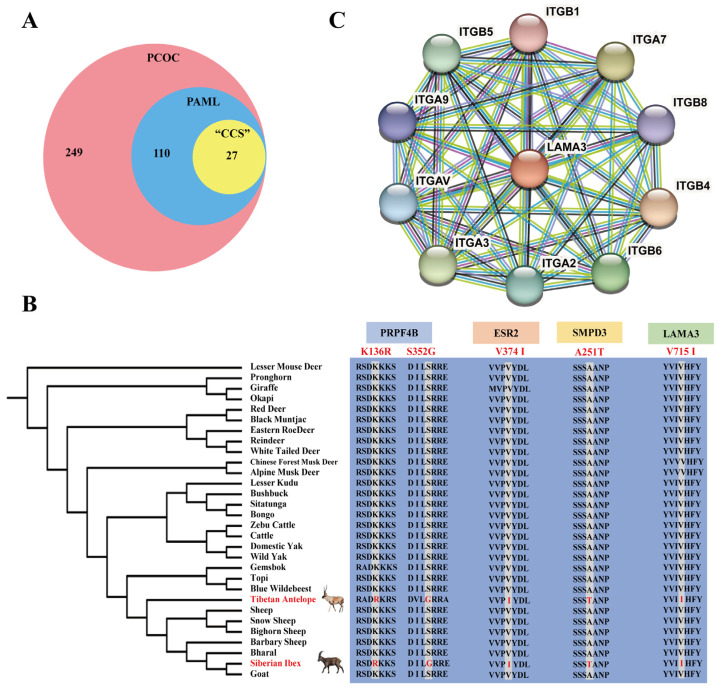
Convergent amino acid substitution genes identified between Tibetan antelope and Siberian ibex. (**A**) Identification of convergent amino acid substitution gene sets using three methods (PCOC: Profile Change with One Change, PAML: Phylogenetic Analysis by Maximum Likelihood, CCS: convergence at conservative sites). (**B**) The convergent amino acid substitution in PRPF4B (Pre-mRNA processing factor 4B), ESR2 (estrogen receptor 2), SMPD3 (Sphingomyelin phosphodiesterase 3) and LAMA3 (Laminin subunit alpha 3) among 30 ruminant species. (**C**) The LAMA3 protein interaction network prediction using STRING online database.

To estimate molecular convergence at amino acid substitution genes in Tibetan antelope and Siberian ibex lineages, we first used the PCOC “Profile Change with One Change” model to detect convergent shifts in amino acid preference. This method would mean a convergent if it occurred toward the same amino acid preference on every branch where the phenotype also changed toward the convergent phenotype [27]. In this method, a total of 386 convergent genes were identified between two lineages (Table S13). At the same time, the “codeml” programs in PAML (Phylogenetic analysis by maximum likelihood) were used to identify the convergent amino acid substitutions; we found 137 convergent genes (Table S14). Combining these two methods, we found that the gene set of the PCOC method was able to fully encompass the PAML results. In addition, in order to reduce false positives and random convergence, we retained only sites where specific mutations occurred in the Tibetan antelope and Siberian ibex lineages using “CCS” (convergence at conservative sites) method [1]. Based on the above method, only 27 convergent amino acid substitution genes were obtained and considered to be highly plausible (Figure 3A and Table S15). For example, PRPF4B (K136R and S352G), ESR2 (V374I), SMPD3 (A251T) and LAMA3 (V715I) show convergent substitution in Tibetan antelope and Siberian ibex (Figure 3B). Among the 27 genes, the KEGG pathway analysis displayed that *LAMA3* can be enriched in the PI3K-Akt signaling pathway, ECM-receptor interaction and focal adhesion simultaneously (Table S16). The *LAMA3* gene belongs to the laminin family and is responsive to several epithelial-mesenchymal regulators, including keratinocyte growth factor, epidermal growth factor and skin fibrosis [28]. Previous studies found that targeted disruption of the *LAMA3* gene causes junctional epidermolysis bullosa in many animals [29,30,31]. Based on this, we hypothesized that *LAMA3* might affect the development of skin hair follicles through these pathways and further regulate cashmere traits. Moreover, we predicted protein–protein interactions of LAMA3 using the STRING database (https://string-db.org/, accessed on 1 December 2022) [32]. The results show that several proteins, such as ITGA2, ITGA3, ITGA9, ITGB1, ITGB4 and ITGB8 had strong interaction with LAMA3 (Figure 3C). These genes are also enriched in PI3K-Akt, ECM-receptor interaction and focal adhesion pathways. On the other hand, we compared the convergent genes with the above PSGs; we found that none of the genes carrying convergent substitutions in Tibetan antelope and Siberian ibex had evolved under positive selection in either species. In summary, the limited evidence suggested convergent environment adaptation under the same positive selection. The convergent evolutionary gene *LAMA3* in Tibetan antelope and Siberian ibex may regulate dermis basement membrane development and cashmere traits through a series of interactions.

### 2.3. Candidate Artificial Selection Genes Screening and Enrichment Analysis of Cashmere Traits

**Figure 4:**
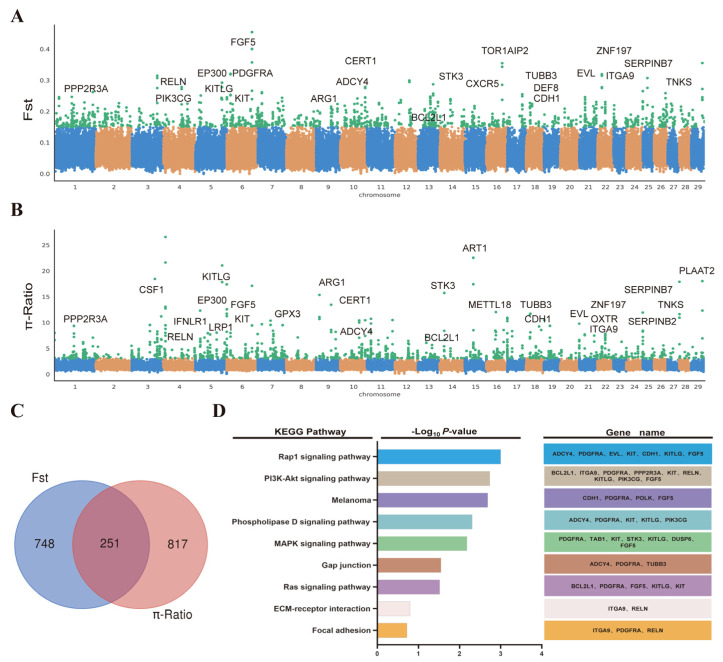
Genome−wide selection sweep for candidate genes in cashmere goats using sliding window analysis. (**A**) Selection signatures in cashmere goats for *Fst*. (**B**) Selection signatures in cashmere goats for π-Ratio. The values were calculated in 50-kb sliding window with 20-kb step across all autosomes, and top 1% values are marked with a green dot. (**C**) The number of shared candidate genes in cashmere goats by two methods. (**D**) KEGG enrichment analysis for the shared candidate genes.

The cashmere traits of cashmere goats underwent strong artificial selection, and some traces of domestication remain in the genome. In order to further explain the influence of artificial selection on cashmere traits, we performed selective sweep analysis (*Fst* and π-Ratio) to detect the selection signatures in the cashmere goat population and non-cashmere goat population based on the whole-genome slide window method. In total, in the top 1% values, we identified 1232 common candidate regions after using *Fst* (*Fst* > 0.1524) and π-Ratio (π-Ratio > 3.0870) (Figure 4A,B and Tables S17 and S18), and annotated 999 and 1068 genes, respectively. We then merged the gene lists generated by these two approaches and identified 251 overlap genes that showed the strongest selection signatures (Figure 4C and Table S19). To obtain a broad overview of the molecular functions of these identified candidate genes for cashmere traits, the KEGG pathway enrichment analysis was performed. Some of the significant KEGG pathways (e.g., Rap1 signaling pathway, PI3K-Akt signaling pathway, MAPK signaling pathway, Ras signaling pathway, and ECM-receptor interaction) were associated with cashmere development. Several candidate genes (e.g., *ADCY4*, *PDGFRA*, *KITLG*, *FGF5*, *BCL2L1*, *PPP2R3A*, *ITGA9* and *RELN*) were also found to be related to the above pathways (Figure 4D and Table S20). In addition, we compared these selected signature genes with PSGs and REGs in Tibetan antelope and Siberian ibex; the number of the shared genes was 4, 2, 15 and 7, respectively (Figure S3). These results suggest that some molecules associated with cashmere traits drove similar phenotypic characteristics under natural and artificial selection conditions.

### 2.4. Identification of Key Gene Modules and Differentially Expressed Genes Related to Cashmere Growth and Development

**Figure 5:**
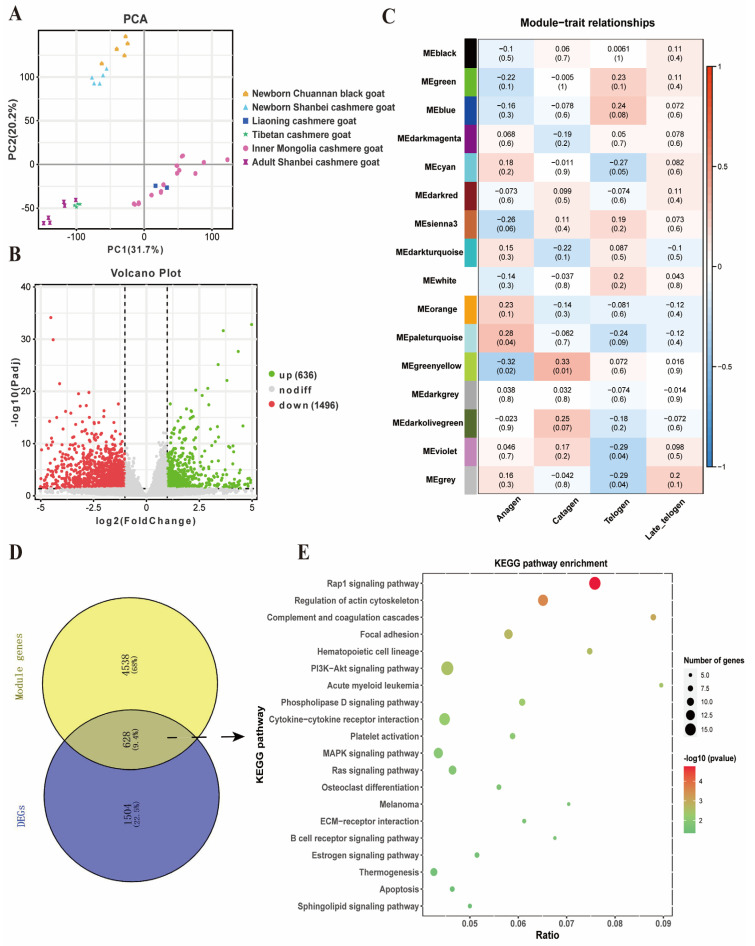
Co−expression analysis of skin transcriptome in cashmere goat. (**A**) The PCA analysis of all skin transcriptome samples of 58 goats from five breeds. (**B**) Volcano plot comparing the change in gene expression of the Chuannan black goats and Shanbei cashmere goats. The green and red dots represent the up-regulated and down-regulated genes, respectively. The gray dots represent genes that are not significantly different in the two breeds (FDR > 0.05). (**C**) Heatmap of the correlation between gene modules and hair follicle cycle features. (**D**) The overlap of differentially expressed genes (DEGs) and two module genes that are closely related to the hair follicle development in anagen. (**E**) KEGG pathway enrichment analysis of 628 shared expression genes.

To further confirm the molecular mechanisms that regulate cashmere traits, we analyzed goat skin transcriptome data. All skin transcriptome samples of 58 goats from five breeds are shown in Figure 5A. Firstly, we used 53 cashmere goat samples for WGCNA analysis. A hierarchical clustering tree for all samples was drawn to identify the outliers in the samples; the results showed that there was no outlier in the samples and that all samples could be used in our subsequent analysis (Figure S4). When the soft-threshold power β = 7, the scale-free network fitting index reached 0.85, which met the scale-free network distribution (Figure S5). Then, the coexpression modules were obtained using the dynamic tree cut method, and similar modules were merged by setting the MEDissThres cutting line to 0.25; a total of 16 modules were screened out (Figure S6 and Table S21). The association between the modules and traits was then analyzed. Two modules ME_paleturquoise_ (cor = 0.28, *p* = 0.04) and ME_greenyellow_ (cor = −0.32, *p* = 0.02), were identified as closely related to the hair follicle development in the anagen (Figure 5C and Figure S7). Secondly, we compared the differentially expressed genes between five newborn Chuannan black goats and five newborn Shanbei cashmere goats. A total of 2132 genes were found to be differentially expressed between Chuannan black goats and Shanbei cashmere goats (|log2FoldChange| > 1.0 and FDR < 0.05). Of these, 636 genes were up-regulated, while 1496 were down-regulated (Figure 5B and Table S22). Then, we integrated the two module genes and differentially expressed genes and found 628 overlapped genes in total (Figure 5D). To investigate potential signaling pathways associated with these shared genes, the KOBAS online software was used for KEGG pathway enriched analysis. The results indicated that these shared genes were mainly involved in the Rap1 signaling pathway, regulation of actin cytoskeleton, focal adhesion, PI3K-Akt signaling pathway, hematopoietic cell lineage, Ras signaling pathway, ECM-receptor interaction and estrogen signaling pathway (Figure 5E and Table S23). Through the gene-pathway network plot (Figure S8A), some hub genes, such as *LAMA2*, *ITGB8*, *ITGA4*, *COL6A6*, *PIK3CA*, *FGF21*, *FGF22* and *CSF1R* were easy to pick out. Moreover, these genes were significantly higher expressed in Shanbei white cashmere goats than in Chuannan black goats (*p* < 0.05) (Figure S8B). Therefore, these genes may be closely related to hair follicle development and the periodic growth of cashmere goats.

### 2.5. Co-Regulatory Network of Cashmere Traits

**Figure 6:**
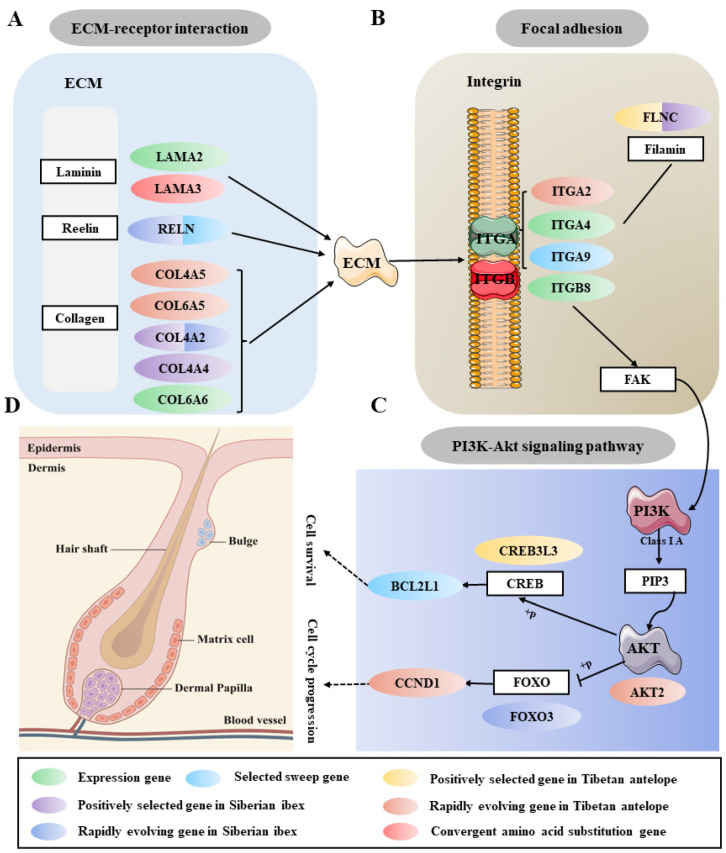
Genes and pathways related to cashmere traits under selection pressure. (**A**) Many extracellular matrix glycoprotein genes in the ECM-receptor interaction pathway may help to maintain cell adhesion and epithelial morphogenesis. (**B**) The integrin α and β subunit (ITGA/ITGB) family genes mediate cell adhesion to the extracellular matrix. (**C**) The genes in the PI3K-Akt signaling pathway are associated with cell survival and cell cycle progression. (**D**) Diagram of hair follicle structure. The results are based on PSGs, REGs, convergent genes, selected candidate genes and expressed genes. Different colors represent different gene sets; two colors indicate that the gene is present in both gene sets.

Cashmere growth is controlled by the hair follicle, a complex skin accessory organ of the goat. The occurrence of hair follicles in the embryonic period and periodic cycle in adulthood involves a series of epidermis and dermis interactions [33]. Therefore, the growth and development of cashmere is a complex biological process regulated by a variety of signaling molecules. According to our results, we found that three KEGG pathways (ECM-receptor interaction, focal adhesion and PI3K-Akt signaling pathway) were commonly enriched in various gene sets (Figure 6). Basement membranes are widely distributed extracellular matrices that coat the basal aspect of epithelial and endothelial cells. This extracellular matrix (ECM) and ECM-associated proteins cooperate to assemble and remodel extracellular matrices and bind to cells through ECM receptors; they could control the survival, proliferation, differentiation, shape, polarity, and motility of cells [34,35]. In this study, we found many extracellular matrix glycoproteins, like Laminin alpha subunits (*LAMA2* and *LAMA3*), Reelin (*RELN*), and type IV collagens (*COL4A2*, *COL4A4*, *COL4A5*, *COL6A5* and *COL6A6*) (Figure 6A). These genes play a significant role in cell adhesion and organ morphogenesis and help maintain normal biological functions. Importantly, we noticed that the *COL6A5* gene occurred rapidly in Tibetan antelope, and its paralog is *COL6A6*, which is differentially expressed in Chuannan black goat and Shanbei white cashmere goat. A recent study found that the *COL6A5* gene may affect cashmere fineness through translatomic analysis in the skin tissue of Liaoning cashmere goats [18]. From this, we can infer that the members of the collagen superfamily (especially *COL6A5* and *COL6A6*) may be important molecular markers affecting cashmere traits.

Focal adhesions are specialized sites of cell attachment to the extracellular matrix (ECM) where integrin receptors link the ECM to the actin cytoskeleton [36]. Integrins are the major family of adhesion molecules that mediate cell adhesion to the extracellular matrix and are composed of α and β subunits heterodimeric receptors [37,38]. We found integrin α and β subunit family genes *ITGA2* (rapidly evolving gene in Tibetan antelope), *ITGA9* (selected sweep gene between cashmere and non-cashmere goats), *ITGA4* and *ITGB8* (high expression genes in cashmere goat skin transcriptome) were strongly enriched in focal adhesion (Figure 6B). In addition, one gene (*FLNC*), the shared positively selected gene between Tibetan antelope and Siberian ibex, was also enriched in this pathway. These results indicate that these signal molecules play an important role in regulating the formation of cashmere. Furthermore, another significantly enriched pathway is the “PI3K-Akt signaling pathway”, which is associated with cell proliferation and apoptosis in a variety of biological processes [39,40]. Highly divergent genes (*CREB3L3*, *AKT*, *FOXO3*, *CCND1* and *BCL2L1*) in this pathway are mainly involved in cell survival and cell cycle progression (Figure 6C), which is important for the growth and development of hair follicles. Together, this evidence demonstrated that adaptive evolution of the above genes associated with epidermis and dermis interactions, cell adhesion, cell survival and cell cycle in Tibetan antelope, Siberian ibex and Cashmere goat had promoted growth and development of hair follicles and the formation of cashmere (Figure 6D). Overall, the multi-omics analysis highlights that the unique features of cashmere traits have led to a series of necessary co-regulatory networks.

## 3. Discussion

With the completion of high-quality genome assembly of more and more species, it is possible to study the adaptive evolution of specific environmental and physiological characteristics of species using comparative genomics methods. Our previously published Ruminant Genome Project de novo assembled genomes of 44 ruminant species, which were better to understand their evolution [13]. For instance, the Chinese water deer (*Hydropotes inermis*) and Forest musk deer (*Moschus berezovskii*) convergent pseudogenization led to secondary loss of headgear [41], the Reindeer (*Rangifer tarandus*) biological adaptation in the Arctic regions [42] and the Giraffes (*Giraffa camelopardalis*) involved in neck elongation and cardiovascular adaptations [43]. In the present study, we aim to assess the evidence for convergent adaptive evolution between Tibetan antelope and Siberian ibex, specifically focusing on cashmere traits. We used the codeml program in the PAML package (version 4.9) [44] to estimate the lineage-specific evolutionary rate for each branch with the phylogenetic. To correlate evidence of convergent evolution under selection pressure, the branch-site model and branch model were used for detecting PSGs and REGs, respectively. We found 10 shared PSGs *(ZNF24*, *FLNC*, *NCOA6*, *CACNG2*, *VPREB1*, *PTRHD1*, *BUB1*, *LOC787891*, *HEYL* and *GJB6*) and 39 shared REGs (e.g., *CCDC187*, *HMBS*, *COL5A3*, *SERPINB9*, *VPREB1*, *DSG1* and *AQP10*) between Tibetan antelope and Siberian ibex. Furthermore, several methods (PCOC model, PAML and “CCS”) have been used to detect adaptive convergent amino acid evolution; we obtained a total of 27 convergent amino acid substitution genes (e.g., *USP24*, *FAM241A*, *ESR2*, *SMPD3*, *PRPF4B* and *LAMA3*) through strict screening conditions. However, none of these genes contained convergent sites and were under positive selection. Davies et al. [7] reported none of the PSGs in all four subterranean mammal lineages for parallel molecular adaptation. Birkeland et al. [8] found that none of the genes carrying convergent substitutions in Arctic branch pairs had evolved under positive selection in both species. In this regard, our conclusion is in line with early studies. Hence, these results demonstrated little evidence for independently fixed mutations at the same sites and positive selection acting on the same genes.

China has famous cashmere goat breeds, such as Inner Mongolia cashmere goat, Liaoning cashmere goat, Tibetan cashmere goat, Shanbei white cashmere goat, etc. Most of these goats live in cold northern regions of China, and their cashmere has undergone intense artificial selection for protection from the cold and as a textile material. Cashmere is produced from the secondary hair follicle of cashmere goat [45], which is finer, softer and lighter, and it is considered a luxury textile fiber [46]. The morphological structure and development of secondary hair follicles directly affect the output and quality of cashmere. Previously studies have identified a large set of genes and pathways that were targets of artificial selection in cashmere goats. For example, Li et al. [24] resequenced 70 cashmere goats and identified 135 genomic regions that were associated with cashmere fiber traits within the cashmere goat populations by analyzing Fst and θπ outlier values. These selected genomic regions contained genes such as *FGF5*, *SGK3*, *IGFBP7*, *OXTR* and *ROCK1*. Gene Ontology enrichment analysis showed that these genes enrichment in keratinocyte differentiation and epidermal cell differentiation. Jin et al. [47] genotyped 53 goats (including Inner Mongolia cashmere goats, Liaoning cashmere goats and Huanghuai goats) using the Illumina Caprine 50K SNP chip and identified some positively selected SNPs by analyzing *Fst* and XP-EHH. Several genes were related to hair follicle development, such as *TRPS1*, *WDR74*, *LRRC14*, *SPTLC3*, *IGF1R*, *PADI2*, *FOXP1*, *WNT10A* and *CSN3*. In this study, we used two methods (*Fst* and π-ratio) to detect the selective sweeps in cashmere goats and non-cashmere goats based on the sliding window method. The results showed that 251 shared genes were found in two methods. The KEGG pathway enrichment analysis showed that these genes could be enriched in the Rap1 signaling pathway, cardiac muscle contraction, PI3K-Akt signaling pathway, Phospholipase D signaling pathway, focal adhesion, Ras signaling pathway, ECM-receptor interaction and calcium signaling pathway. Then, we overlapped these genes with PSGs and REGs and obtained four (*LRP1*, *TADA2B*, *OXTR*, *TNKS*), two (*ARG1*, *ZNF197*), 15 (e.g., *PIK3CG*, *KITLG*, *LRP1*, *RRR22*, *CXCR5*, *ARG1*, *TNKS*, etc.) and seven (*LSS*, *MCM3AP*, *YBEY*, *RELN*, *KIT*, *NLRC4*, *ARHGAP33*) shared genes in Tibetan antelope and Siberian ibex, respectively (Figure S3). We found that some genes undergoing positive selection and rapid evolution under natural environmental pressure could be repeated in the selection signals of artificially bred cashmere goats. In a word, the evidence presented above suggests that in Tibetan antelope and Siberian ibex, as the different lineages expanded into similarly cold environments millions of years ago, natural selection targeted some of the same genetic loci with cashmere traits that would later be targets of artificial selection for the breeding of cashmere goats in cold regions of north China.

In order to further focus on the convergent molecular characteristics of cashmere traits in Tibetan antelope, Siberian ibex and cashmere goats during natural and artificial selection, we studied the gene expression patterns of skin transcriptome in cashmere goats. Studies have shown that the secondary hair follicle of cashmere goats undergo an obvious periodic cycle in a year [33,48]. Thus, it is important to explore the mechanism of hair follicle development and cycle in order to improve cashmere performance. To do this, we integrated 53 skin transcriptome data of cashmere goats (including Inner Mongolia cashmere goats, Liaoning cashmere goats, Tibetan cashmere goats and Shanbei white cashmere goats) to explore the gene coexpression network and identify hub genes closely related to each stage of the hair follicles cycle using WGCNA method. After constructing the weighted gene coexpression network modules and correlating them with the hair follicle development stages (divided into anagen, catagen, telogen and late-telogen), two key modules (ME_paleturquoise_ and ME_greenyellow_) containing 5166 genes were identified as closely related to the hair follicle development in anagen. Moreover, we compared gene expression between Chuannan black goat and Shanbei white cashmere goat and obtained 2132 differentially expressed genes. We overlapped these two module genes and differentially expressed genes and found 628 coexpression genes. These genes are mainly enriched in the Rap1 signaling pathway, regulation of actin cytoskeleton, focal adhesion, PI3K-Akt signaling pathway, hematopoietic cell lineage, Ras signaling pathway, ECM-receptor interaction and estrogen signaling pathway. Our results are consistent with previous reports, especially for similar functional pathways [49,50].

Therefore, integrating these multi-omics analysis results, it is not difficult to find that some genes and pathways regulating cashmere traits are common. We found ECM-receptor interaction, focal adhesion and PI3K-Akt signaling pathway were common enriched pathways in a variety of gene sets (Figure 6). During these three pathways, we found many convergent molecular features. The convergent amino acid substitution gene (*LAMA3*), positively selected genes in Tibetan antelope (*FLNC*, *CREB3L3*), positively selected genes in Siberian ibex (*FLNC*, *COL4A2*, *COL4A4*), rapidly evolving genes in Tibetan antelope (*COL4A5*, *COL6A5*, *ITGA2*, *AKT2*, *CCND1*), rapidly evolving genes in Siberian ibex (*RELN*, *COL4A2*, *FOXO3*), selected signature genes of cashmere goat (*RELN*, *ITGA9*, *BCL2L1*) and coexpression genes in skin transcriptome of cashmere goat (*LAMA2*, *COL6A6*, *ITGA4*, *ITGB8*) were enriched in convergent similar functional pathways among natural and artificial selection. Although previous studies on cashmere traits have also found these three signaling pathways to varying degrees, the regulatory network relationship between them has not been systematically and completely clarified. To the best of our knowledge, we first used convergent evolution, selection signatures and gene expression to explore the cashmere traits, and combined with multiple gene sets, we summarized the network relationships among multiple pathways that regulate cashmere traits. Nevertheless, it has to be said that these specific molecular functions need to be confirmed by further experiments.

## 4. Materials and Methods

### 4.1. Species Selection and Data Collection

Based on our previous Ruminant Genome Project [13], we first selected 30 ruminant species genomes spanning six families: *Tragulidae* (*n* = 1), *Antilocapridae* (*n* = 1), *Giraffidae* (*n* = 2), *Cervidae* (*n* = 5), *Moschidae* (*n* = 2) and *Bovidae* (*n* = 19). Detailed genome assembly information is shown in Table S1 and downloaded from Ruminant Genome Database RGD2.0 (http://animal.nwsuaf.edu.cn/code/index.php/RGD, accessed on 10 December 2022) [51]. These genomic data were used for comparative genomic analysis. In addition, we collected total of 156 samples of whole-genome resequencing data, including 117 non-cashmere and 39 cashmere goats. The raw data were downloaded from the NCBI database (https://www.ncbi.nlm.nih.gov/, accessed on 1 January 2022) and our previously published research [52]. Detailed information about all samples in this study was provided in Table S2. Our dataset also contained transcriptome sequencing information from skin tissues of cashmere goats in different stages of hair follicle development. Raw data were downloaded from the Sequence Read Archive (SRA) database of the NCBI. A total of 48 samples were collected, including Tibetan Cashmere goats (*n* = 4), Inner Mongolia Cashmere goats (*n* = 36), Shanbei Cashmere goats (*n* = 6) and Liaoning Cashmere goats (*n* = 2). The samples’ detailed information is shown in Table S3. In addition, we sequenced ten newborn goats’ skin tissues using RNA-seq, including Chuannan black goat (*n* = 5) and Shanbei white cashmere goat (*n* = 5). The species information and detailed data are listed in Table S4 and Figure S1.

### 4.2. Phylogenetic Tree and Multiple Sequence Alignment

First of all, we aligned the genome sequences of 30 ruminant species to the cattle (*Bos taurus*) genome using LAST (version last867) [53] with the following parameters: “lastal-m100-E0.05”. Then we used MULTIZ (version 11.2) [54] to merge all the multiple alignment files (MAF) into the multiple sequence alignment results. The orthologous genes were identified using the synteny blocks in the multiple sequence alignment results. All of the 30 species’ whole-genome phylogenetic trees were constructed using the fourfold degenerate site (4DTv) of one-to-one orthologous genes and were extracted using in-house Perl scripts. The phylogenetic tree was constructed using IQ-TREE (version 1.6.6) [55] software with the following parameters: “-nt 4-bb 1000 -m TEST” and visualized with iTOL [56] software.

### 4.3. Analysis of Positively Selected Genes and Rapidly Evolving Genes

We used a conserved genome synteny methodology to establish a high-confidence orthologous gene set in 30 ruminant species. To find genes undergoing specific adaptations in Tibetan antelope (*Pantholops hodgsonii*) and Siberian ibex (*Capra sibirica*), we applied the codeml package in the PAML (version 4.9e) software [44] with a free-ratio model (model = 1) and estimated lineage-specific evolutionary rates for each branch. Based on the results, we further analyzed the rapidly evolving genes (REGs) and positively selected genes (PSGs) using a branch model (model = 2) and branch-site model (model = 3), respectively. An LRT (likelihood ratio test) was performed to compare a model that allows sites to be under positive selection on the foreground branch with the null model in which sites may evolve either neutrally or under purifying selection. We then calculated the *p*-value for each gene based on the Chi-square statistics, and the genes with *p*-values of less than 0.05 were treated as candidates for the two gene sets.

### 4.4. Identification of Convergent Amino Acid Substitutions

The multiple sequence alignments of orthologous proteins of ruminants allowed us to search for convergent amino acid substitutions at a molecular level. Generally, a convergent substitution is defined as changes resulting in the same amino acid at the same site in two lineages, and divergent substitutions as changes resulting in different amino acids at the same site in two lineages [57]. In the present study, we first used a PCOC (“Profile Change with One Change”) model [27] to look for convergent shifts in amino acid preferences between Tibetan antelope and Siberian ibex. The PCOC > 0.9 and OC > 0.9 were used as screening criteria to define the convergence sites. Subsequently, the ancestral amino acid sequences were reconstructed for 14,013 orthologous genes with at least 75% valid amino acid information with the codeml program in PAML v4.9e [44]. Convergent sites were identified if an amino acid residue changed at the same site in the Tibetan antelope and Siberian ibex lineages and were different from that of their respective most recent common ancestor. Finally, in order to filter out noises as stringently as possible, we applied the “convergence at conservative sites” (CCS, for short) method [1] to detect convergence. This method is also known as “identical”, meaning that the same amino acid substitution occurs at the same site only in two convergent lineages and is different from other lineages. In general, we have obtained a relatively reliable convergent amino acid substitution gene set in Tibetan antelope and Siberian ibex by combining various approaches.

### 4.5. Reads Alignment and SNP Calling

The resequencing clean reads from all individuals were aligned to the latest goat reference genome using Burrows–Wheeler Aligner (BWA-MEM v0.7.15) with default settings [58]. Duplicate reads were filtered using the “REMOVE_DUPLICATES = true” option in Picard v2.1 (https://broadinstitute.github.io/picard/, accessed on 1 May 2022). The “HaplotypeCaller”, “GenotypeGVCFs”, and “SelectVariants” programs in the Genome Analysis Toolkit (GATK version 3.8) [59] were used for calling raw SNPs. All SNPs were filtered using the “Variant Filtration” module of GATK with the standard parameters as below: Variants with QD < 2.0, FS > 60.0, MQ < 40.0, MQRankSum < −12.5, ReadPosRankSum < −8.0 and SOR > 3.0. Last, only biallelic SNPs identified by GATK that all show no more than 10% missing data were extracted using VCFtools (version 0.1.15) [60] with “--min-alleles 2 --max-alleles 2 --max-missing 0.9.” All VCF files of each sample were used for selective sweep analysis.

### 4.6. Genome-Wide Selective Sweep Test

To identify the selective sweep regions, the population fixation index (*Fst*) and nucleotide diversity (θπ) were estimated based on a sliding window approach with windows of 50 kb and a step of 20 kb. The *Fst* was calculated using VCFtools [60] with the parameter “--weir-fst-pop group1 --weir-fst-pop group2 --fst-window-size 50,000 --fst-window-step 20,000 --maf 0.05 --max-missing 0.90”. The π-Ratio was calculated using VCFtools with parameters “--keep group1/group2 --window-pi 50,000 --window-pi step 20,000 --maf 0.05 --max-missing 0.90”. Due to the power of each method being different, any set of candidate genes may contain some false positives. In this study, the overlap of the top 1% windows of the two methods was considered as candidate signatures of selection. The functional annotation of associated SNPs was carried out according to the goat reference genome using the ANNOVAR package v10.23.2012 [61].

### 4.7. RNA Sequencing Data Analysis

The raw data were first converted into fastq format by NCBI SRA toolkit (version 3.0.0) software (https://github.com/ncbi/sra-tools, accessed on 1 January 2022) and used fastp software [62] to filter adaptor sequences and low-quality reads. Then the clean reads were mapped to the goat (*Capra hircus*) reference genome ARS1(GCF_001704415.1) using HISAT2 [63]. Transcript assembly and standardized FPKM (Fragments per kilobase of transcript per million) value were obtained using StringTie (Version 2.1.2) [64] to construct the gene expression matrix. The negative binomial generalized linear models of DESeq2 [65] were used to compare the expression levels of the samples from five Chuannan black goats and five Shanbei white cashmere goats. Genes with |log2FoldChange| > 1.0 and false discovery rate (FDR) value < 0.05 were defined as significant differentially expressed genes (DEGs).

### 4.8. Construction of Weighted Gene Coexpression Network

The weighted gene co-expression network analysis was performed using WGCNA, an R software package [66], which was built in R 4.0.2 using RStudio (http://www.rstudio.org, accessed on 1 May 2022). First of all, the expression matrix was standardized FPKM value, and genes with expression standard deviations (SDs) of less than 0.5 were removed in each sample. The samples were clustered to construct the sampleTree, and detected outliers were selected based on cut height. Then, to achieve a scale-free network, the pickSoftThreshold function was used to select an appropriate soft threshold power β by calculating the adjacency between genes. Finally, in order to identify gene modules, the adjacency matrix was transformed into the topological overlap matrix (TOM), and a hierarchical clustering tree was constructed according to the corresponding dissimilarity (dissTOM = 1-TOM) with the minModuleSize 30. The genes with similar expression patterns were clustered into the same module by the module eigengenes (MEs). The modules with more than 75% similarities were merged (MEDissThres = 0.25).

### 4.9. Identification of Significant Modules and Key Genes Related to Hair Follicle Development

According to previous reports, the hair follicle development of cashmere goats shows periodic changes in 12 months of the year [33]. We divided the development of hair follicles into four stages: anagen (April–September), catagen (October–December), telogen (January and February) and late-telogen (March). In an effort to visually represent the relationships between modules and the phenotypic traits of hair follicle development, Pearson’s correlation coefficient was calculated and plotted in a heatmap. The highest correlating module was chosen as the key module for further analysis. In the present study, we focused on modules and genes associated with cashmere development and growth, so the key modules in anagen were selected. The module membership (MM) indicated the correlation between the gene expression profile and each ME. The gene significance (GS) was used to describe the correlation between MEs and hair follicle cycle traits. In order to screen out hub genes based on GS and MM values, genes with the highest MM and highest GS in modules of interest were considered candidates. Thus, the |GS| > 0.2 and |MM| > 0.8 were set as cut-off criteria to screen genes in the key module with high functional significance.

### 4.10. Gene Enrichment Analysis

The online tool KOBAS-i (version 3.0) (http://kobas.cbi.pku.edu.cn/, accessed on 1 October 2022) [67] was used to identify the significantly enriched Kyoto Encyclopedia of Genes and Genomes (KEGG) pathways for PSGs, REGs, convergent genes, selected candidate genes and significantly expressed genes set. Significance was calculated using the hypergeometric test/Fisher’s exact test, and a *p*-value < 0.05 was considered.

## 5. Conclusions

In conclusion, this study adopted a more novel and comprehensive approach to investigating cashmere traits. Our results found some molecular characteristics of convergent cashmere traits in Tibetan antelope, Siberian ibex and Cashmere goat, especially type IV collagen family genes (*COL4A2*, *COL4A4*, *COL4A5*, *COL6A5*, *COL6A6*) and integrin family genes (*ITGA2*, *ITGA4*, *ITGA9*, *ITGB8*), and revealed the similarly convergent signal pathways (ECM-receptor interaction, focal adhesion and PI3K-Akt signaling pathway) between natural and artificial selection. These findings provide some valuable references for the adaptive evolution of cashmere and broaden the molecular basis for cashmere goat breeding.

## Data Availability

The raw RNA-seq data of goat skin tissues that support the findings of this study have been deposited into the CNGB Sequence Archive (CNSA) of China National GeneBank DataBase (CNGBdb) with accession number CNP0003532 and also at Sequence Read Archive (SRA) database of National Center for Biotechnology Information (NCBI) database with accession number PRJNA885592.

## Supplementary Materials

The following supporting information can be downloaded at: https://www.mdpi.com/article/10.3390/ijms24021165/s1.
