# Supplementary material for: Convergent Genomic Signatures of Cashmere Traits: Evidence for Natural and Artificial Selection
Source: Int J Mol Sci. 2023 Jan 6;24(2):1165. doi: 10.3390/ijms24021165 (PMC9860930; doi:10.3390/ijms24021165)
Supplement: Supplementary file 1 [file ijms-24-01165-s001.zip › Supplementary Figures.pdf]

Supplementary Materials

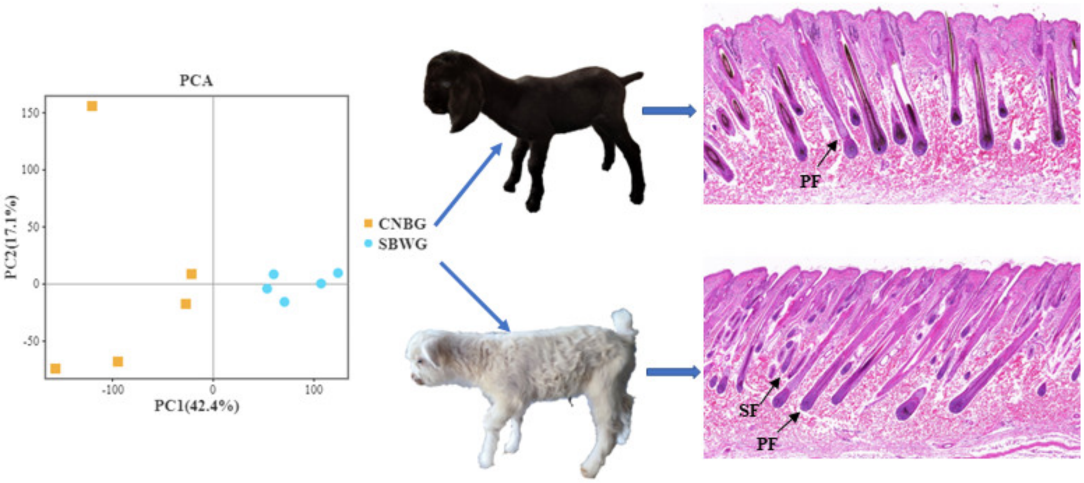

Figure S1 Samples information. CNBG: Chuannan black goat (n=5); SBWG: Shanbei white cashmere goat (n=5); PF: Primary follicles; SF: Secondary follicles.

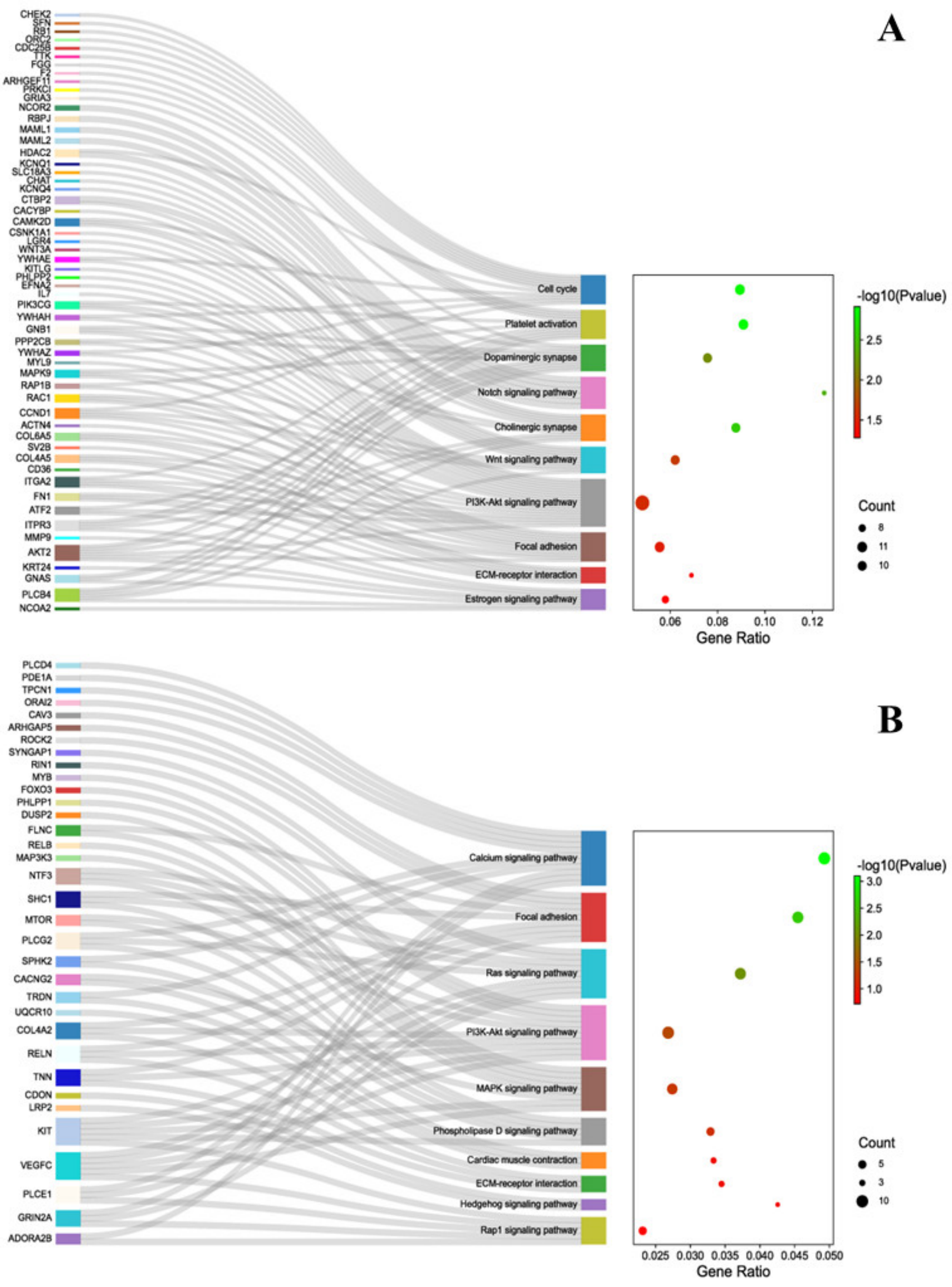

Figure S2 The KEGG enrichment analysis of rapidly evolving genes in Tibetan antelope and Siberian ibex. **(A)** The result of rapidly evolving genes in Tibetan antelope. **(B)** The result of rapidly evolving genes in Siberian ibex. On the left is the Sankey diagram, which represents the genes contained in each pathway; on the right is the dot plot, where the dot size represents the number of genes and the dot color represents the *P* value.

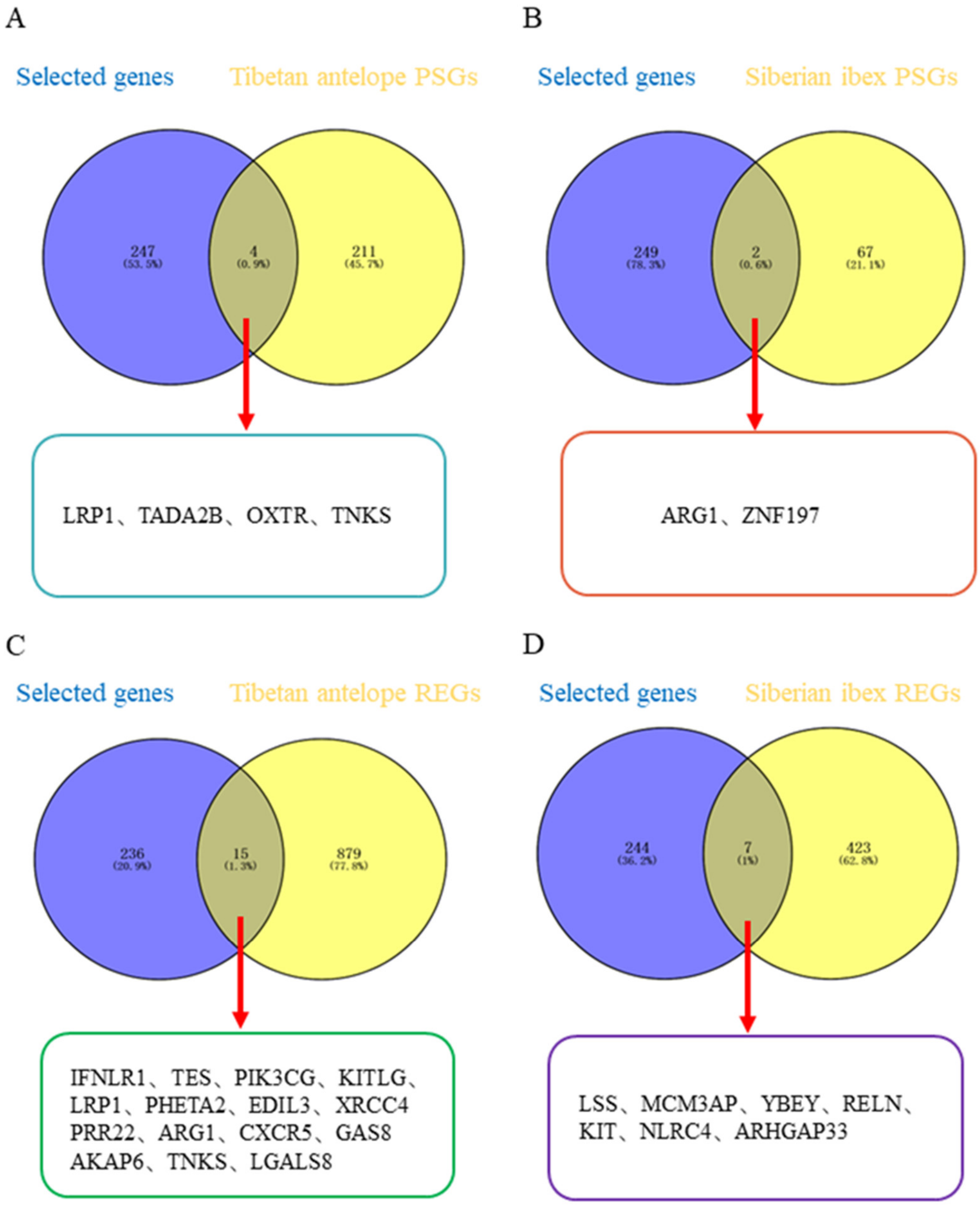

Figure S3 Genes shared by natural and artificial selection. **(A)** The overlap of selected genes and Tibetan antelope PSGs. **(B)** The overlap of selected genes and Siberian ibex PSGs. **(C)** The overlap of selected genes and Tibetan antelope REGs. **(D)** The overlap of selected genes and Siberian ibex REGs.

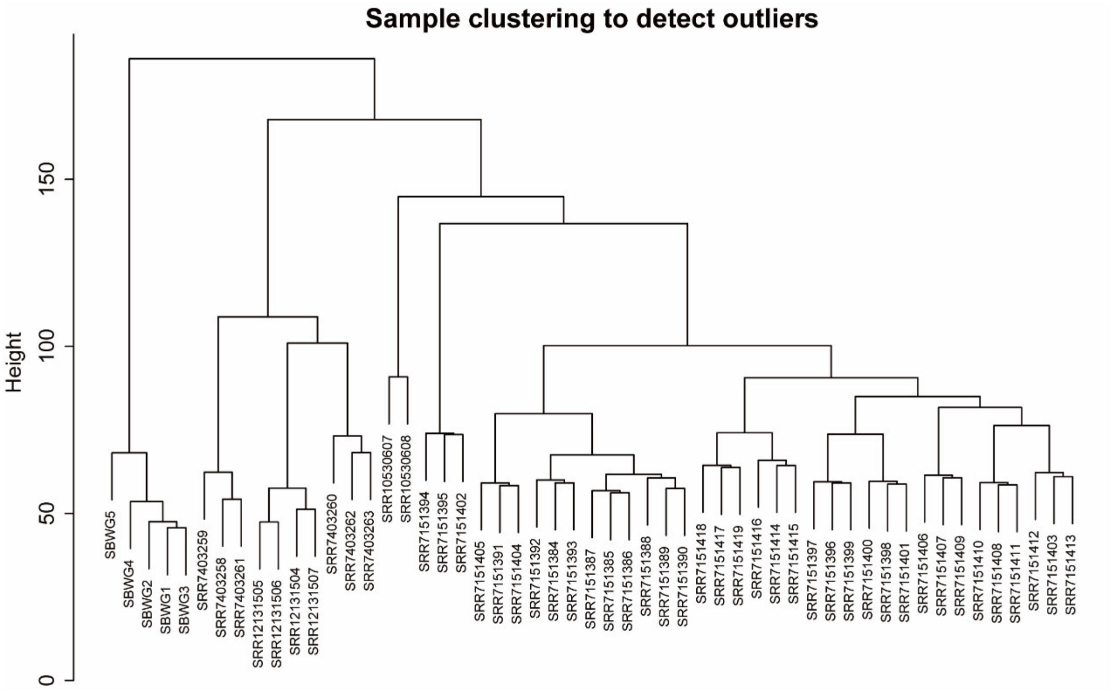

Figure S4 Hierarchical clustering information of 53 cashmere goat samples.

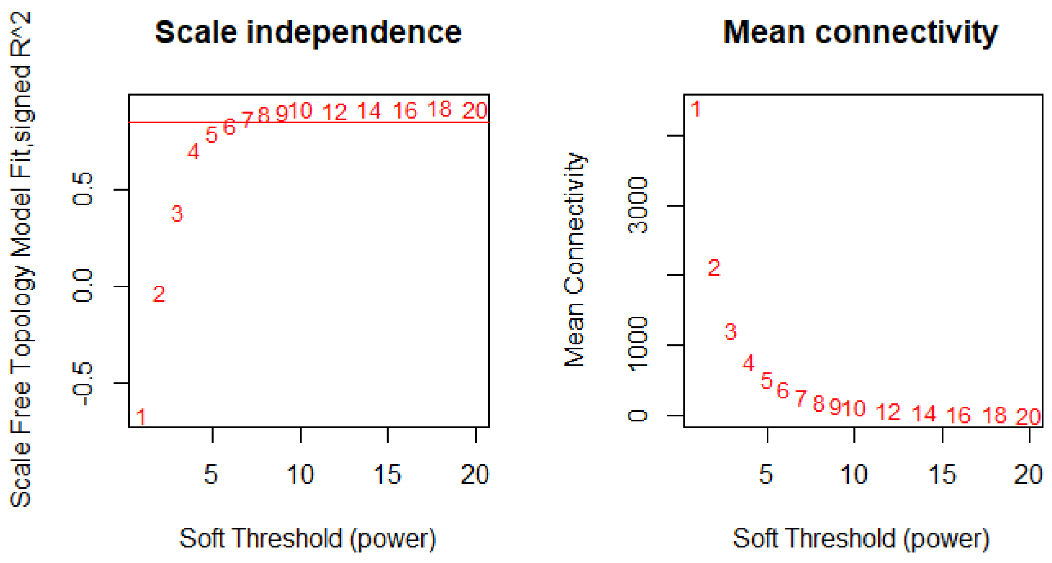

Figure S5 The determination of soft thresholding power. Left figure is a scale-free fitting index responding to various soft-thresholding powers. Right figure represents the mean connectivity of different soft-thresholding power. The approximate scale-free topology can be obtained at the soft-thresholding power of 7.

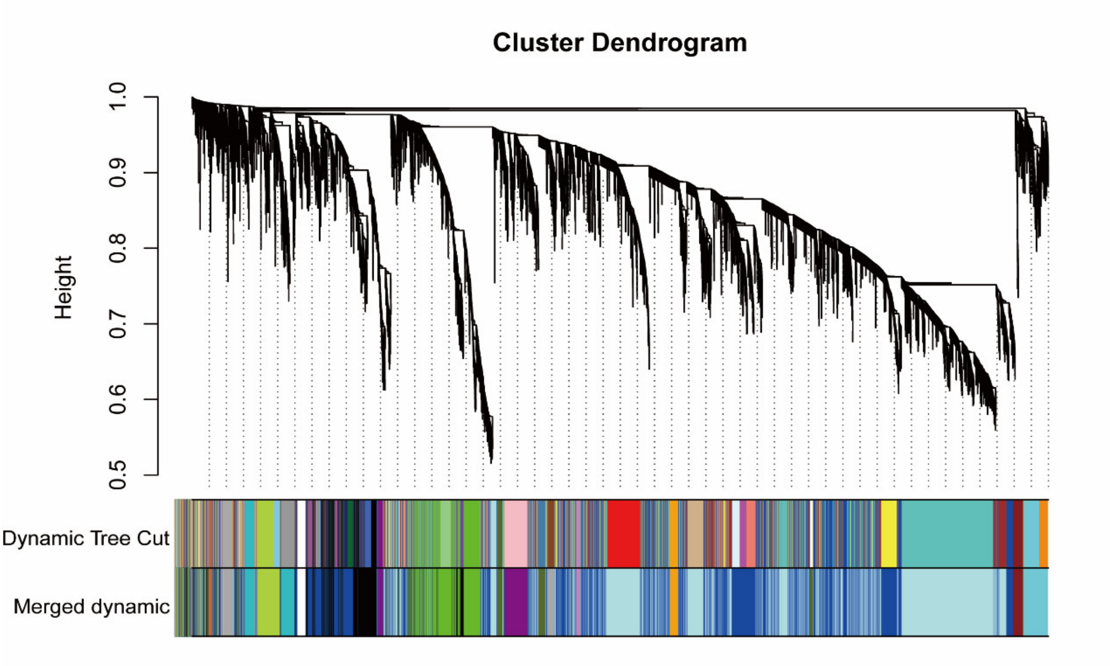

Figure S6 The gene cluster dendrogram was constructed according to hierarchical clustering of adjacency-based dissimilarity. The each of color blocks represent gene co-expression modules by the dynamic hybrid-cutting method, and similar modules were merged by setting the MEDissThres cutting line to 0.25 (i.e., the models with 75% of eigengenes similarity were merged).

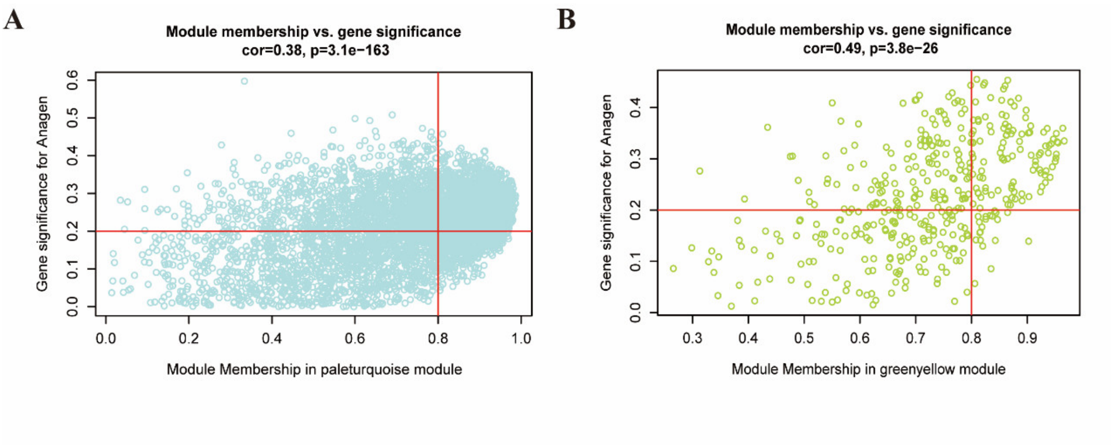

Figure S7 The correlation between genes in corresponding modules and gene expression profiles was defined as module membership (MM) and gene significant (GS). **(A)** paleturquoise module. **(B)** greenyellow module. The thresholds of cor. gene MM > 0.8 and cor. gene GS > 0.2 were established to screen key genes in each module.

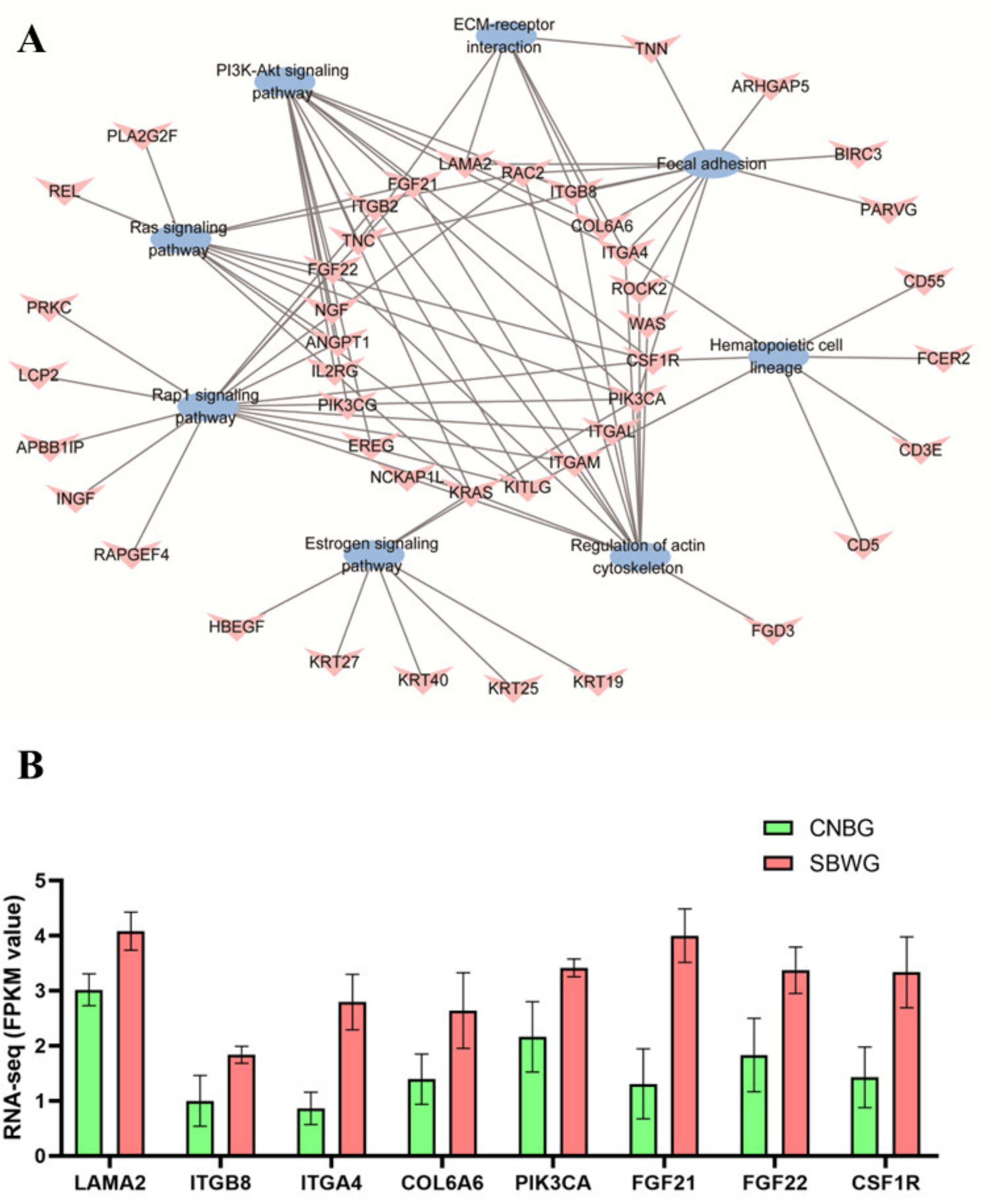

Figure S8 Gene expression regulatory network. **(A)** Gene-pathway network. **(B)** Gene expression levels of some hub genes in Chuannan black goats and Shanbei white cashmere goats. CNBG: Chuannan black goat; SBWG: Shanbei white cashmere goat.
